# Role of suture anchors in management of fractures of inferior pole of patella

**DOI:** 10.4103/0019-5413.65149

**Published:** 2010

**Authors:** Ashish Anand, Manish Kumar, Gautam Kodikal

**Affiliations:** Centre for Joint Replacement, Wockhardt Hospitals, Bangalore-560 076, India

**Keywords:** Anchor sutures, fracture patella, inferior pole fractures of patella

## Abstract

**Background:**

The traditional recommendation for displaced comminuted inferior pole fractures is excision of the comminuted pole followed by reattachment of the patellar tendon with transosseous suture. To the best of our knowledge there has been no previous published study mentioning the use of suture anchors for fracture inferior pole of patella. We present a retrospective analysis of five cases of patients doing well at final follow-up of two years.

**Materials and Methods:**

Five patients treated at our institute using suture anchors for repair of comminuted inferior pole fractures of patella between January 2007 to March 2007. (range 28 years-55 years). There were three males and two females.

**Results:**

The average follow-up was 25 months (range 24 months-26 months). The patients were evaluated for range of motion, strength, patellofemoral scores and any alteration of patellar height. The outcome of the procedure was assessed with use of the patellofemoral scoring system of Noyes *et al*,^5^ as adapted by Saltzman *et al*.^6^ The final patellofemoral score (maximum 100 points) was 94.6 (range 93-96).

**Conclusion:**

We believe it is a novel extended indication of the use of suture anchors and should be in the armament of every trauma surgeon.

## INTRODUCTION

The incidence of inferior pole fractures of patella is around 5% and the various treatment options include tension band wiring, circumferential wiring, or use of screw.^1^–^3^ The traditional recommendation for displaced comminuted inferior pole fractures is excision of the comminuted pole followed by reattachment of the patellar tendon with transosseous suture. There has been one published study on better outcomes obtained following use of a basket plate to attach the avulsed inferior pole of patella as compared to resection of inferior pole of patella^4^. To the best of our knowledge there has been no previous published study mentioning the use of suture anchors for fractures of the inferior pole of patella.

## MATERIALS AND METHODS

This retrospective study includes five patients treated by using suture anchors for repair of comminuted inferior pole fractures of patella between January 2007 to March 2007. All the patients had presented within a few hours after the injury to the hospital. The mechanism of injury was a direct fall on the knee. None of the patients had any other concomitant injury to the ipsilateral limb and none of the fractures were compound. All the patients were operated within 24 hours following the injury. An informed consent was taken from all the patients.

### Operative procedure

A pneumatic tourniquet was used in all cases and prior to inflating the tourniquet, 1 gram of cefuroxime was given intravenously. A standard midline exposure (4 cm incision) extending from the middle of the patella to the upper end of tibial tuberosity was used. Fracture was identified and irrigated with saline and the haematoma was removed. Comminuted inferior pole pieces were excised. *Two suture anchors Twin Fix ti (Smith-Nephew, Ma, USA) of size 3.5 mm*, each loaded with 2 Ultra braid sutures were placed in the center of the large proximal piece. The distance between the two anchors was 1 cm. With the help of free Mayo needle the ultrabraid was then threaded through the proximal part of the patellar tendon and held under tension. Intra operative X-rays were taken to ensure that there was no tilting of the patella in the saggital plane and knots were tied in the standard fashion. Care was taken to avoid any over tensioning of the knot which would have resulted in tilting of the patella in the saggital plane. All the patients had varying degrees of retinacular tears which were repaired with O vicryl. Knee flexion was checked at this time. The average flexion achieved on table was 95 degrees (range 95°-100°) and the fixation was stable at this angle. Wound was irrigated and closed in the standard fashion.

Patients received two more doses of antibiotics in the postoperative period (eight hours and 16 hours after the completion of the procedure).

Patients were placed in knee immobilizer in full extension for four weeks to allow for healing of retinaculum repair as well as allowing healing of ligament to bone. X-rays were done at first postoperative day. The length of patella and the patellar tendon as measured on the lateral view was recorded. Patients were discharged on the second postoperative day. Sutures were removed at two weeks.

In the immediate postoperative period patients were encouraged to do isometric Quadriceps exercises - straight leg exercises and ankle pumps. No knee motion was allowed for the first four weeks. Patients were allowed weight bearing as tolerated with the help of crutches on knee immobilizer. The immobilizer was discontinued and range of motion, quadricep strengthening and hamstring strengthening exercises were started at four weeks following surgery. At the same time crutches were discontinued. All the exercises were done under the direct supervision of a physiotherapist.

## RESULTS

**Figure 1:**
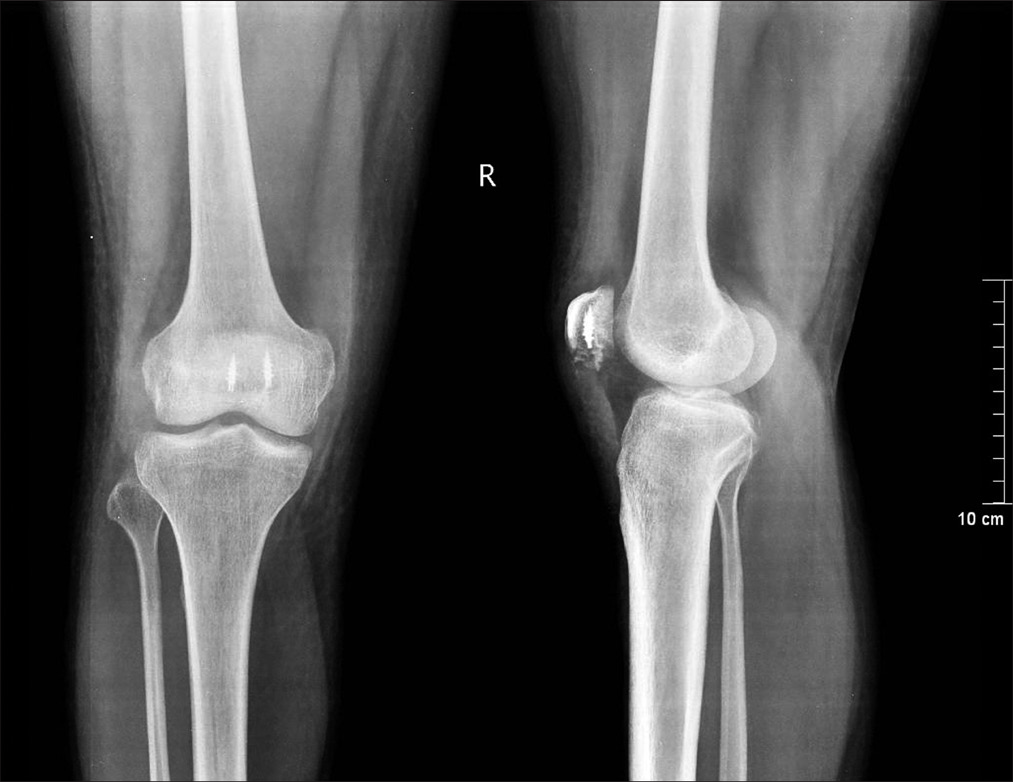
X-rays (anteroposterior and lateral) at final follow- up showing anchors in place and maintained patellar height

**Figure 2:**
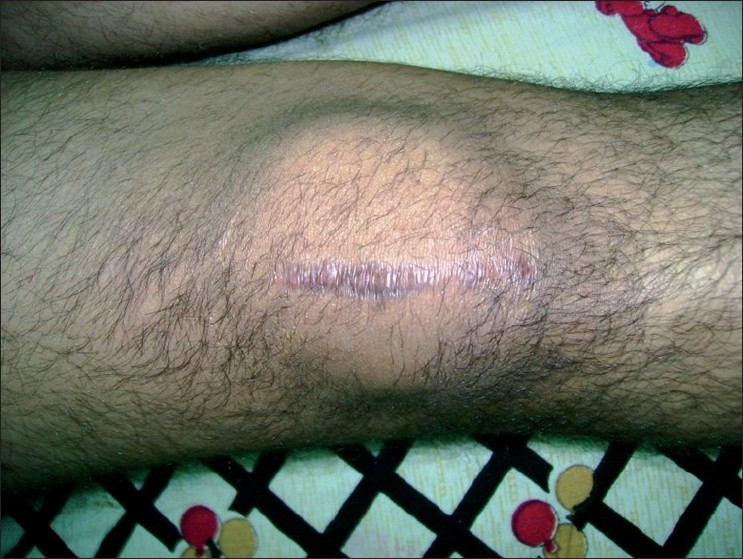
Clinical photograph showing surgical scar mark and full extension

**Figure 3:**
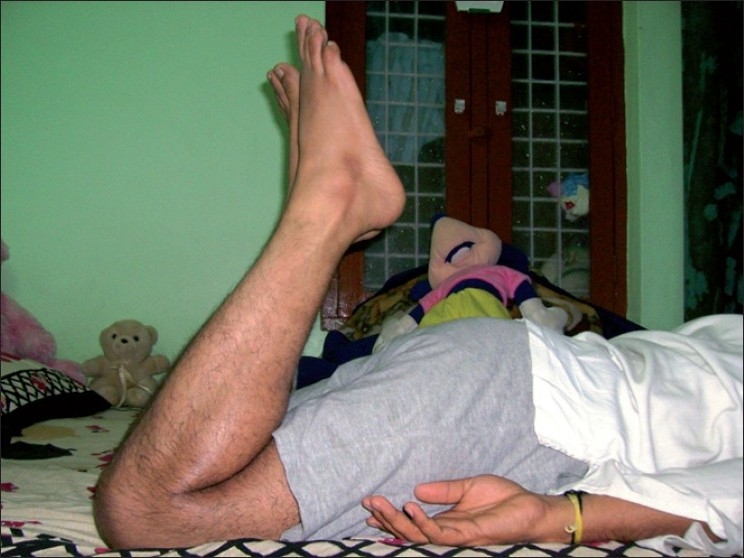
Clinical photograph showing comparable flexion

The average follow-up age of the patients was 30.2 years (range 28-55 years). There were three males and two females. There were no postoperative infections. The average was 25 months (range 24 months-26 months). The patients were evaluated for range of motion, strength, patellofemoral scores and any alteration of patellar height. The outcome of the procedure was assessed with use of the patellofemoral scoring system of Noyes *et al*,^5^ as adapted by Saltzman *et al*.^6^ To address problems of the patellofemoral joint, evaluation involved the completion of a questionnaire (maximum score, 45 points), a clinical evaluation (maximum score, 43 points), and a radiographic analysis (maximum score, 12 points). The overall score was rated as excellent (90 to 100 points), good (80 to 89 points), fair (70 to 79 points), or poor (<70 points). The patients had a final mean range of movement 130° which was comparable to the other side. The time required to recover full range of motion following discontinuation of immobilizer was 3 weeks (range 2.5-4.5 weeks). The time required to achieve strength (as checked by the dynamometer) comparable to the other side was four months (range four to five months) following surgery. X-rays were done at three months, six months, one year and two years after surgery [Figure 1]. None of the X-rays revealed any alteration of patellar height (same length of patella and patellar tendon as measured on the lateral view) or any evidence of tilting of the patella at final follow up. None of the patients had any flexion deformity or extensor lag at three months and at final follow up [Figures 2 and 3] the final patellofemoral score (maximum 100 points) was 94.6 (range 93-96).

## DISCUSSION

The use of suture anchors was initially used for Rotator cuff repairs but gradually the indications have expanded. Anchors are impacted in cancellous bone of the tuberosity of the humerus and the preloaded sutures are shuttled through the rotator cuff. The suture material used is non-absorbable and composed of a special ultra high molecular weight (UHMW) polyethylene fiber and features a unique braid configuration. As a result, ultrabraid suture offers special advantages over traditional polyester suture, including higher knot breaking strength, increased lubricity, and a stronger resistance to fraying. This is preloaded on to TwinFix suture anchor which has two ultrabraid sutures.

The site of reattachment of the patellar ligament following partial patellectomy is a controversial area with some^6^,^7^ proposing attachment near the articular surface while others^8^ advocating attachment near the anterior cortex. We chose to insert the anchors in the center of the proximal remnant as this would have theoretically the least chance of causing the tilt in the patella and also give us a maximum purchase in the cancellous bone (thickest area of the bone).

It is important to protect the repair because the powerful forces are generated by the quadriceps mechanism. This is usually accomplished by figure of eight, load sharing wire or cable.^9^ The cable protects the patellar tendon repair by transmitting loads directly from the Quadriceps tendon or proximal pole of the patella to the tibial tubercle. The disadvantage of using cable wire is that they create additional stress risers in the Patella and the Tibial Tubercle. Secondly they usually require removal one to two years after surgery.

In the technique used by us we did not require the use of the cable wire for protection of the repair. This might be attributed to the superior strength of the suture material (Ultrabraid) and also the protective effect of the external knee immobilizer. The immobilizer was used to allow for healing of the retinacular tears as well as healing of the patellar ligament to the bone.

Veselko *et al*,^4^ have reported good long term results with basket plate osteosynthesis versus patellar ligament repair for inferior pole avulsion patellar fractures. Patellar height is preserved at long term follow-up and mobilization is much faster as contrast with ligament repair. In the Vasalko *et al*. study none of the cases were comminuted^4^ and they did not use bone anchors. Although mobilization was delayed in our cases there was no compromise on the final outcome. Patellar height was preserved at last follow up with comparable range of motion.

To the best of our knowledge, there has been no previous documented use of suture anchor for fracture patella. Part of it may be attributed to the absence of concrete laboratory data which can confirm its use for comminuted fracture of inferior pole of patella. Theoretically inserting the suture anchors in cancellous bone without rim of cortex could be a weak point where the repair might fail. We did not observe any such failures. The size of the distal fragment which was excised was less than 20% of the size of the patella in all the cases. There is no previous published data on this issue, however we think this is the recommended upper limit.

The limitations of this study are: it is a retrospective review of cases which theoretically lends a bias, less number of cases and absence of controls. More laboratory studies and randomized control trials are required to validate our point.

We believe it is a novel extended indication of the use of suture anchors and should be in the armament of every trauma surgeon.
